# Identification of Dynamic Recrystallization Model Parameters for 40CrMnMoA Alloy Steel Using the Inverse Optimization Method

**DOI:** 10.3390/ma18030718

**Published:** 2025-02-06

**Authors:** Xuewen Chen, Qiang Li, Bingqi Liu, Shiqi Zhao, Lei Sun, Hao Yi

**Affiliations:** School of Materials Science and Engineering, Henan University of Science and Technology, 263 Kaiyuan Avenue, Luoyang 471023, China; 220320020227@stu.haust.edu.cn (Q.L.); lbq8565@163.com (B.L.); 230410020020@stu.haust.edu.cn (S.Z.); sunlei7870@163.com (L.S.); bayue33368@163.com (H.Y.)

**Keywords:** dynamic recrystallization, inverse optimization method, 40CrMnMoA alloy steel, adaptive simulated annealing algorithm

## Abstract

The microstructure of 40CrMnMoA during hot forging determines its macroscopic mechanical properties. Dynamic recrystallization (DRX) behavior is commonly used to refine grains and improve the microstructure of materials; therefore, it is important to be able to predict mechanical behavior during hot forging and the microstructure evolution during dynamic recrystallization. In order to accurately determine the DRX model parameters of 40CrMnMoA steel, an inverse optimization method is proposed in this work. The uniaxial isothermal compression experiment of 40CrMnMoA steel was carried out on a Gleeble-1500D thermal simulation tester (Dynamic Systems Inc. (DSI), Poestenkill, NY, USA) under the temperature range of 900~1200 °C and the strain rate range of 0.005 to 5 s^−1^. Based on the true stress–strain data obtained by a compression test, the DRX model of 40CrMnMoA was initially established using the traditional averaging method. Subsequently, the DRX model parameters calculated by the conventional averaging method were used as the initial values, the mean-square error between the experimental and calculated values of the DRX volume fraction was set as the objective function, and the DRX model parameters were optimized by the adaptive simulated annealing (ASA) algorithm. By comparing the correlation coefficient *R*, average absolute relative error (*AARE*), and the root mean square error (*RMSE*) of the predicted DRX percentage with the experimental values before and after optimization, it was found that the optimized model achieved an *R*-value of 0.992, with *AARE* and *RMSE* decreased by 34% and 2%, respectively, which verified the accuracy of the optimized DRX model. Through the program’s secondary development, the optimized DRX model of 40CrMnMoA was integrated into finite element software Forge^®^ 3.2 to simulate the isothermal compression process. The comparison between grain size from the central region of simulation results and actual samples revealed that the relative error is less than 3%. This result demonstrated that the inverse optimization method can accurately identify the DRX model parameters of 40CrMnMoA alloy steel.

## 1. Introduction

As society continues to progress, the annual production of cement is huge, and the demand for energy-saving technology is growing [1]. The roller press, as a new type of cement grinding equipment, came into being [2]. Extrusion rollers, as key parts of the roller press, have mechanical properties that largely determine the service life of the roller press [3]. The alloy steel 40CrMnMoA, as a new type of extrusion roll material, is favored for its high strength, high hardness and excellent wear resistance, and is widely used in the manufacture of various rolls. However, due to the large size of extrusion rollers and unreasonable hot forging technology, microstructural defects, such as coarse grains and mixed crystals, will inevitably occur during the manufacturing process, thus reducing the performance of the extrusion rollers and seriously shortening the service life of the roller press [4]. Therefore, improving the mechanical properties of extrusion rolls has become a key challenge in manufacturing roller presses. An in-depth study of the deformation behavior and organization evolution of 40CrMnMoA alloy steel at high temperatures is essential to improve the forging process of extrusion rolls. By optimizing the production process, the internal grain structure of the extrusion roll can be effectively refined, thus significantly improving its mechanical properties. This not only enhances the durability of the extrusion roll, but also extends the overall service life of the roller press, which is important for improving production efficiency and reducing maintenance costs.

Dynamic recrystallization (DRX) is a crucial softening mechanism in metallic materials during the hot forging process, which helps to eliminate work hardening, re-refine grains and improve the microstructure and properties of materials. This process typically occurs during plastic processing of metallic materials, such as hot rolling and forging, where materials are subjected to high temperatures and stresses. As a result, the original grain structure is disrupted and reorganized into new, finer grains [5]. Microstructural evolution models enable us to analyze the DRX behavior of metallic materials during deformation processes quantitatively. As a result, the recrystallization behavior of a wide variety of materials has been intensively studied by many researchers around the world with fruitful results. To quantitatively characterize the interrelationship between alloy dynamic recrystallization and deformation parameters like strain levels, Sellars et al. [6] first proposed a grain size prediction model of C-Mn steel in 1979 to predict the grain size of C-Mn steel during hot deformation. Although it is a semi-empirical model, it has clear physical significance, easily determinable parameters, and a certain degree of versatility. Yada et al. [7] further developed the Yada dynamic recrystallization model in 1982, which included strain and was used to predict the DRX percentage during the material forming process. Kim et al. [8,9], McQueen [10], and Kopp et al. [11,12] conducted research on different materials and proposed different microstructural evolution models, respectively. Among these, the Yada and Kopp DRX models have explicit parameters that are easy to determine and have been widely applied. Subsequently, Chen et al. [13] established a DRX kinetic model for the 30Cr2Ni4MoV alloy, analyzing its thermal deformation behavior and characterizing the evolution of the DRX microstructure. Fang et al. [14] established a critical strain and DRX volume fraction model for the FGH96P/M superalloy to predict its DRX behavior. Shang et al. [15], based on tensile tests of 316LN stainless steel, constructed a dynamic recrystallization model for this material and predicted its recrystallization process across various temperatures and strain rates.

DRX is divided into two main types: continuous dynamic recrystallization (CDRX) and discontinuous dynamic recrystallization (DDRX) [16,17]. CDRX predominantly occurs in materials with high stacking fault energy, such as aluminum and iron. In contrast, DDRX occurs more often in materials with low to medium stacking fault energies. Numerous scholars have conducted extensive research on the impact of deformation parameters on DRX mechanisms. Babu et al. [18] investigated the intricate relationship between DRX of 304H austenitic stainless steel and initial grain size and strain rate. It was found that under low strain rates, both CDRX and DDRX occur, while at high strain rates, CDRX is predominant. Wang et al. [19] studied the effect of strain rate and twin on the flow behavior and microstructure evolution of type 304 austenitic stainless steel, and determined that the alloy underwent DDRX and CDRX at a low strain rate and a high strain rate, respectively. Li et al. [20] employed the Zener–Hollomon (Z) parameter to delve into the microstructure and mechanisms of recrystallization evolution in the Al–Mg–Si alloy across various LnZ conditions. The results demonstrated that the deformed microstructure is closely related to the Z value, with the subgrain size and the DRX volume fraction increasing as the LnZ parameter decreases. Zhang et al. [21] investigated the DRX mechanisms of the 2195 aluminum alloy and discovered that DDRX predominates at intermediate temperatures, while CDRX is more prevalent at higher temperatures, which suggests that temperature markedly affects the recrystallization mechanism in aluminum alloys. Jia et al. [22] conducted high-temperature compression experiments on the Inconel 625 alloy under various conditions and found that CDRX with subcrystalline progressive fusion and alloy rotation plays a dominant role at low temperatures (<1100 °C), while at high temperatures (≥1100 °C), DDRX with twin-induced bulging boundaries plays a dominant role. Jeong et al. [23] investigated DRX mechanisms of the Al0.7CoCrFeMnNi high-entropy alloy. They concluded that CDRX is primary for both the FCC and BCC phases. However, the kinetics of CDRX in the BCC phase are faster, leading to a higher proportion of DRX grains in the BCC phase under the same deformation conditions.

However, the uneven distribution of the temperature field and equivalent strain field during the forging process of extrusion rollers makes it difficult to predict and control their recrystallization behavior and grain size. Utilizing the finite element analysis (FEA) method, the developed DRX model can be integrated into the FEA software to simulate the hot forging process of metallic materials. This allows for direct visualization of the DRX volume fraction of metals under various conditions. An accurate DRX model is the prerequisite for precise numerical simulation. Therefore, proposing a new method for accurately determining the DRX model parameters of 40CrMnMoA alloy steel and combining this DRX model with finite element software is of significant importance for addressing the issues of coarse and mixed grains in extrusion rollers.

In the existing literature, the establishment of DRX models mostly adopts the traditional averaging method, while the global inverse optimization method is rarely reported. The traditional averaging method is a very convenient way to determine the model parameters. This method first takes the natural logarithm of the model, then plots a scatter diagram under a certain condition with known parameters, and performs linear fitting on the scatter diagram. The intercept or slope obtained is the required parameter. Subsequently, the parameters obtained under different conditions are averaged as the model parameters. In fact, the parameters calculated under different deformation conditions vary greatly. Averaging these parameters is merely a compromise, with significant differences, sometimes even differing by several orders of magnitude. The calculated parameters are not the optimal solution. In contrast, the inverse optimization method can comprehensively consider the effect of deformation parameters on the model under different conditions, and optimize the model as a whole according to the determined objective function, which can find the optimal solution and obtain a more accurate model.

This work aims to fill the gap in the existing literature in understanding the hot deformation behavior of 40CrMnMoA alloy steel. Based on true stress–strain data obtained from a Gleeble-1500D thermal simulation machine (Dynamic Systems Inc. (DSI), Poestenkill, NY, USA), a new inverse optimization method was proposed to establish the Kopp DRX model. First, the model was built using the traditional averaging method, and then the model was optimized using global inverse optimization technology to improve the prediction accuracy of the model. To further verify the effectiveness of the optimized DRX model, it was embedded into the finite element software Forge^®^ 3.2 through the program’s secondary development to simulate the hot compression process, and then the grain size in the central region of simulation results was compared with those of actual specimens.

## 2. Materials and Methods

**Figure 1:**
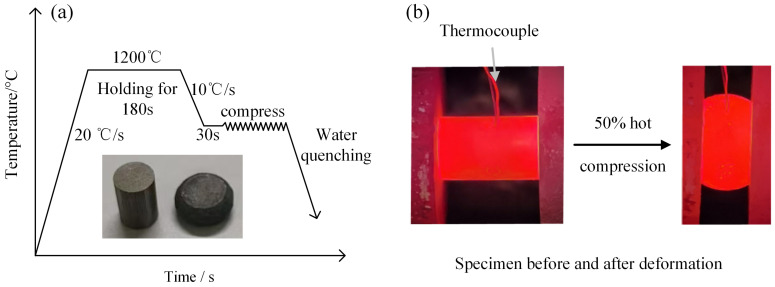
(**a**) Compression test scheme of 40CrMnMoA steel; (**b**) The specimen changes during the compression test.

In this study, 40CrMnMoA alloy steel (Provided by Citic Heavy Machinery Co., Ltd., Luoyang, China), intended for the manufacture of extra-large extrusion rollers, was investigated, with its chemical composition presented in Table 1. Compression specimens, measuring Ø8 × 12 mm, were prepared. Tests were carried out on a Gleeble-1500D thermal simulation tester (Dynamic Systems Inc. (DSI), Poestenkill, NY, USA) according to the protocol shown in Figure 1. The specimens were heated to 1200 °C at a rate of 20 °C/s, held for 180 s, then cooled to the test temperature at a rate of 10 °C/s and held for 30 s. Hot compression tests were performed under strain rates of 0.005, 0.01, 0.1, 1, and 5 s^−1^, at temperatures ranging from 900 to 1200 °C, with a 50% compression reduction. Following deformation, the specimens were water-quenched to preserve the microstructure. The compressed cylindrical specimens were axially cut, embedded, ground with sandpaper, polished with diamond abrasive paste, and etched in a 60 °C water bath with a supersaturated picric acid solution for 15 min. The etched specimens were observed using a Zeiss Axio Vert A1 metallurgical microscope (Beijing Precision Instrument Co., Ltd., Beijing, China), and microstructural images of 40CrMnMoA steel under different deformation conditions were obtained.

## 3. Results and Discussion

### 3.1. True Stress–Strain Curves

**Figure 2:**
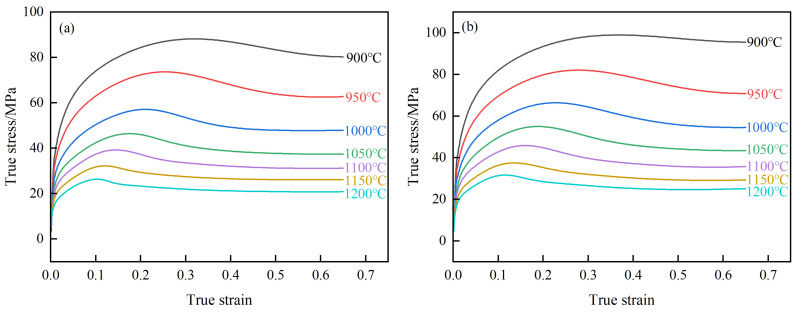

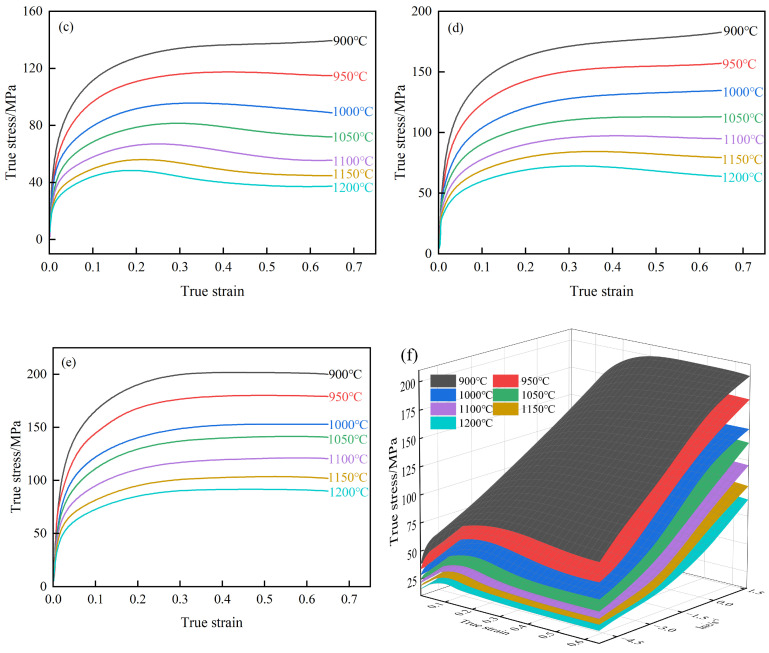
True stress–strain curves of 40CrMnMoA steel under various deformation conditions: (**a**) 0.005 s^−1^; (**b**) 0.01 s^−1^; (**c**) 0.1 s^−1^; (**d**) 1 s^−1^; (**e**) 5 s^−1^; (**f**) 3D response surface graph of true stress–strain.

Figure 2 illustrates the true stress–strain curves of 40CrMnMoA alloy steel under a range of deformation conditions. Observably, the stress exhibits an initial rise followed by a decline in response to increasing strain, particularly at lower strain rates. For example, under the condition of 0.005 s^−1^, 900 °C, the stress of the steel first reaches the peak stress of 88 MPa, then gradually decreases to 80 MPa and stabilizes, exhibiting the typical characteristics of a DRX curve. This phenomenon stems from the effects of work hardening. Initially, as deformation commences, the swift proliferation and interaction of dislocations in metal amplify the resistance they encountered during movement, resulting in a rapid increase in flow stress. With the deformation of metal, the dynamic recovery (DRV) effect is intensified, slowing down the increased rate of flow stress. Upon reaching the critical strain, DRX occurs within the metal; the metal is simultaneously influenced by the combined effect of DRV and DRX. The stress value gradually rises to a peak and then begins to decline. Ultimately, the stress stabilizes when the combined effect of DRV and DRX offset work hardening. This transformation illustrates the complex interaction of microstructure changes on the mechanical response of materials. At high strain rates, this characteristic exists only at high temperatures. For instance, under the condition of 5 s^−1^, 1200 °C, the stress of 40CrMnMoA alloy steel initially reaches a peak stress of 91 MPa, then gradually decreases to 90 MPa. This is due to the fast deformation rate, which results in the short nucleation time and incomplete dynamic recrystallization. In addition, the stress reduction in this case is relatively small. At low temperatures and elevated strain rates, the stress of 40CrMnMoA alloy steel exhibits a gradually increasing trend. For example, at the condition of 5 s^−1^, 900 °C, the flow stress climbs progressively alongside strain, peaking at 202 MPa, beyond which it levels off, indicative of a DRV curve characteristic. At the outset, stress escalation is swift with strain escalation, a consequence of work hardening. With ongoing deformation, DRV mitigates the hardness of the material, decelerating the flow stress’s ascent until it plateaus.

Additionally, with constant temperature and strain, the true stress of 40CrMnMoA alloy steel escalates with the acceleration of the strain rate. This escalation is attributed to intensified dislocation interactions at higher strain rates, which amplify the resistance to deformation. Conversely, with fixed strain rate and strain, the true stress of the 40CrMnMoA alloy diminishes as the temperature ascends. This reduction is ascribed to the heightened thermal activation energy at elevated temperatures, which invigorates atomic motion and expands the array of operational slip systems, thereby contributing to the alleviation of true stress.

In order to visually observe the relationship between temperature, strain, strain rate and stress, a three-dimensional response surface graph is used, as shown in Figure 2f. This graph clearly shows how these three variables work together to affect stress. It can be seen that the stress increases and then decreases with the increasing strain, and shows different trends at different strain rates and temperatures. Overall, the higher the temperature, the lower the strain rate and the lower the stress. This graphical approach helps to quickly identify key factors that affect material properties and guide material selection and process optimization.

### 3.2. Analysis of DRX Structure Evolution

**Figure 3:**
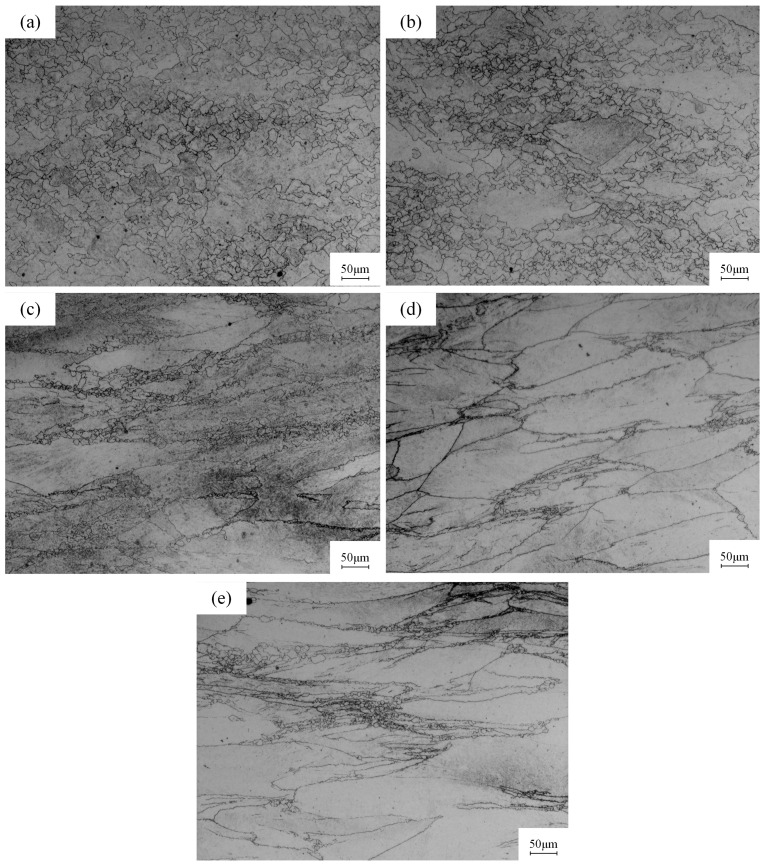
Microstructure of 40CrMnMoA alloy steel at different strain rates at 900 °C: (**a**) 0.005 s^−1^; (**b**) 0.01 s^−1^; (**c**) 0.1 s^−1^; (**d**) 1 s^−1^; (**e**) 5 s^−1^.

Figure 3 presents the metallographic images of specimens at different strain rates at 900 °C. It can be seen that at low strain rates, the presence of numerous fine, equiaxed grains along grain boundaries is evident, signifying DRX in the metal. With increasing strain rate, grain nucleation decreases, leading to larger grain sizes. Metallographic analysis indicates that the average grain size of metal is 14.599 μm at 900 °C, 0.005 s^−1^, and it increases to 53.557 μm at 900 °C, 5 s^−1^. This is attributed to the continuous rise in dislocation density within the metal during low strain rate deformation, coupled with dislocation climb and cross-slip, which culminate in new grain nucleation and DRX. The stress of the metal begins to decrease due to DRV and DRX softening effects. Consequently, the stress–strain curve demonstrates the characteristic of increasing initially and then decreasing, which is the typical feature of a DRX curve [24]. In contrast, at high strain rates, the brief deformation duration limits nucleation opportunities, resulting in minimal or no DRX. The metal is subject to the effects of work hardening and DRV, causing the stress–strain curve to exhibit a sustained ascent, which is characteristic of a typical dynamic recovery curve [25].

**Figure 4:**
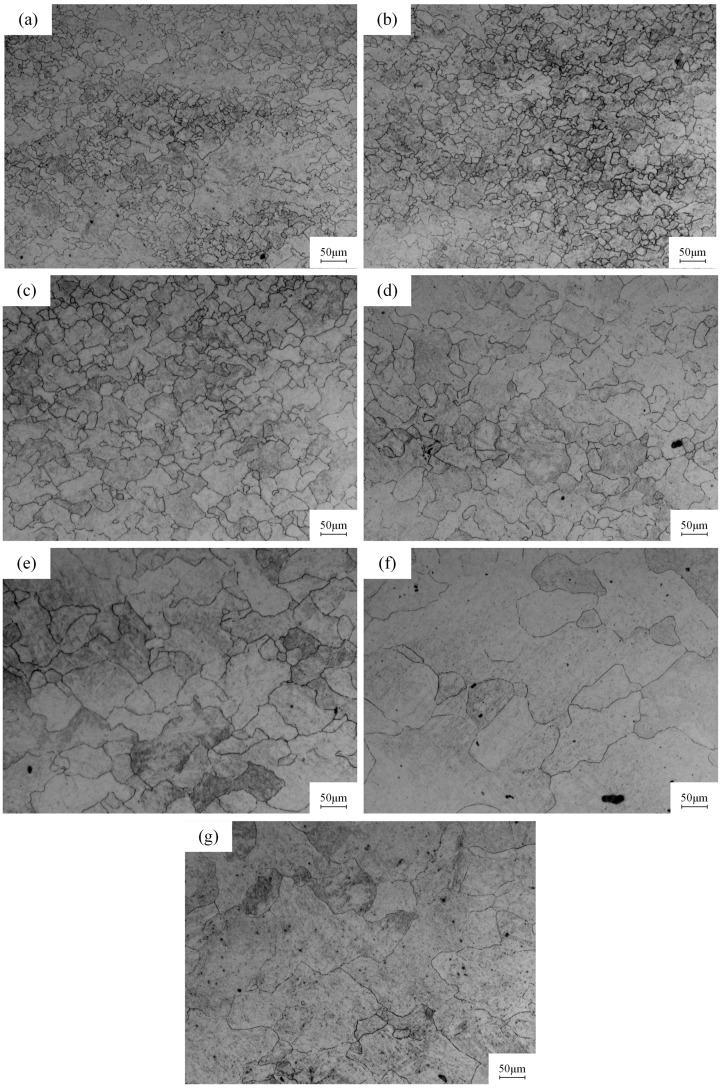
Microstructures of 40CrMnMoA alloy steel at different temperatures under 0.005 s^−1^ strain rate: (**a**) 900 °C; (**b**) 950 °C; (**c**) 1000 °C; (**d**) 1050 °C; (**e**) 1100 °C; (**f**) 1150 °C; (**g**) 1200 °C.

Figure 4 shows the metallographic photos of specimens at different temperatures under 0.005 s^−1^ strain rate. At 900, 950, 1000, 1050, 1100, 1150 and 1200 °C, the grain sizes are 14.599, 17.385, 25.951, 30.222, 47.608, 61.708 and 53.751 μm, respectively. This indicates that at the same strain rate, with the increase in temperature, the grain size of the metal gradually increases and the strength gradually decreases [26], which can be intuitively seen in Figure 4. The reason behind this is the escalation of the internal activation energy of metals with temperature, rendering the metal more susceptible to DRX. Consequently, the fine grains nucleated by DRX are more inclined to grow at elevated temperatures, leading to an increase in grain dimensions.

### 3.3. Establishment and Validation of DRX Model for 40CrMnMoA Steel

#### 3.3.1. Acquisition of Critical Strain and Peak Strain Parameters

**Figure 5:**
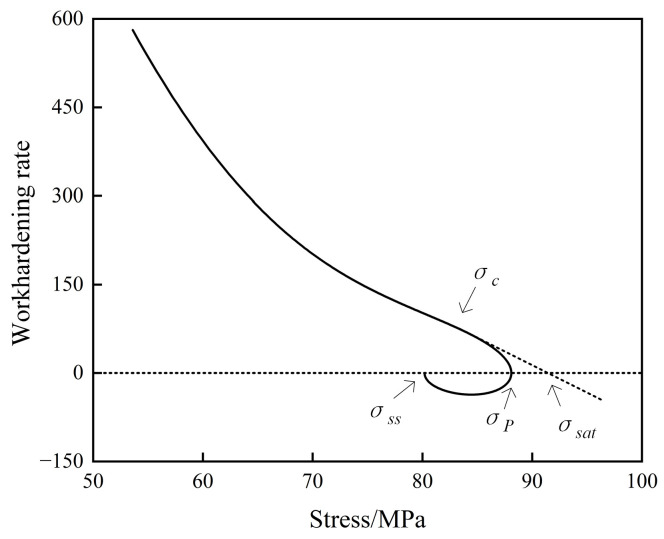
Work-hardening rate–stress curve of 40CrMnMoA alloy steel at 005 s^−1^, 900 °C.

In the metal hot forming process, DRX is triggered once the strain surpasses a threshold value, referred to as the critical strain. During the deformation process, the dislocation density within the metal continuously increases, providing more driving force for the metal to undergo DRX behavior. Once the metal deformation reaches the critical strain, new grains begin to form at grain boundaries, and the metal undergoes DRX [27]. Figure 5 shows the work-hardening rate–stress curve of 40CrMnMoA alloy steel at 005 s^−1^, 900 °C, from which relevant information of dynamic recrystallization, such as critical stress $\;\sigma_{c}$, peak stress ${\;p}_{c}$, saturation stress ${\;\sigma }_{\mathrm{sat}}$ and steady-state stress ${\;\sigma }_{\mathrm{ss}}$, can be obtained.

Because the true stress–strain curve cannot intuitively represent the critical strain value, Poliak and Jonas [28] introduced a novel approach to ascertain the critical strain for the initiation of DRX in metals. This method sidesteps the need for extrapolated flow stress data, identifying the onset of DRX by scrutinizing the inflection point in the θ−σ curve, where *θ* represents the work-hardening rate, that is, the derivative of stress with respect to strain (dσ/dε). This technique allows for a precise determination of the critical strain and stress conditions that signal the commencement of DRX, providing a clearer understanding of the behavior of materials under various thermal deformation conditions. The stress and work-hardening rate corresponding to the inflection point are the critical stress ($\sigma_{c}$) and the critical work-hardening rate ($\theta_{c}$), respectively.

The inflection point is the point where $\partial^{2}\theta /\partial \sigma$ = 0, Since θ = ∂σ/∂ε [29], this can be obtained:

(1) $$\frac{\partial \theta }{\partial \sigma }=\frac{\partial \theta }{\partial \varepsilon }\cdot \frac{\partial \varepsilon }{\partial \sigma }=\frac{1}{\theta }\cdot \frac{\partial \theta }{\partial \varepsilon }=\frac{\partial (\ln\theta )}{\partial \varepsilon }$$

Hence, lnθ−ε curves are charted under various conditions, utilizing a cubic polynomial for curve fitting. Subsequently, the resulting fitted function is subjected to differentiation.

(2) $$\ln\theta =A+B\varepsilon +C\varepsilon^{2}+D\varepsilon^{3}$$

(3) $$\partial (\ln\theta )/\partial \varepsilon =B+2C\varepsilon +3D\varepsilon^{2}$$

The extremum points on the curve correspond to the critical strain points.

(4) $$\varepsilon_{c}=-\frac{C}{3D}$$

Consequently, the critical strains $\varepsilon_{c}$ for various conditions were derived, as depicted in Table 2.

#### 3.3.2. Establishment of DRX Model Using the Averaging Method

The method of analyzing true stress–strain curves was chosen to determine the DRX percentage of 40CrMnMoA alloy steel at strain rates 0.005 s^−1^, 0.01 s^−1^, and 0.1 s^−1^ with different temperatures. At the same time, the dynamic recrystallization phenomenon was not obvious at 0.1 s^−1^, 900 °C, so it was not considered. According to the research of Laasraoui et al. [30], the correlation between DRX volume fraction ($X_{\mathrm{drx}}$) and stress of metals during hot deformation is depicted in Equation (5):

(5) $$X_{\mathrm{drx}}=\frac{\sigma_{\mathrm{drv}}-\sigma }{\sigma_{\mathrm{sat}}-\sigma_{\mathrm{ss}}}$$

where $\sigma_{\mathrm{drv}}$ signifies the true stress value considering only DRV, that is, the stress corresponding to the strain on the DRV curve of metals; $\sigma_{\mathrm{sat}}$ denotes the saturated stress of dynamic recovery; $\sigma_{\mathrm{ss}}$ represents the steady-state stress of the true stress-strain curve. As stated in the study [31], the DRV curves under various conditions can be formulated by Equation (6):

(6) $$\sigma_{\mathrm{drv}}=\sigma_{\mathrm{sat}}+(\sigma_{c}-\sigma_{\mathrm{sat}})\exp\left[\frac{(\varepsilon -\varepsilon_{c})\theta_{c}}{\sigma_{c}-\sigma_{\mathrm{sat}}}\right]\sigma_{c}\leq \sigma \leq \sigma_{\mathrm{sat}}$$

Drawing from the θ−σ curves of 40CrMnMoA steel across various conditions, $\sigma_{\mathrm{sat}}$, $\sigma_{\mathrm{ss}}$, $\sigma_{c}$, and $\theta_{c}$ were determined.

**Figure 6:**
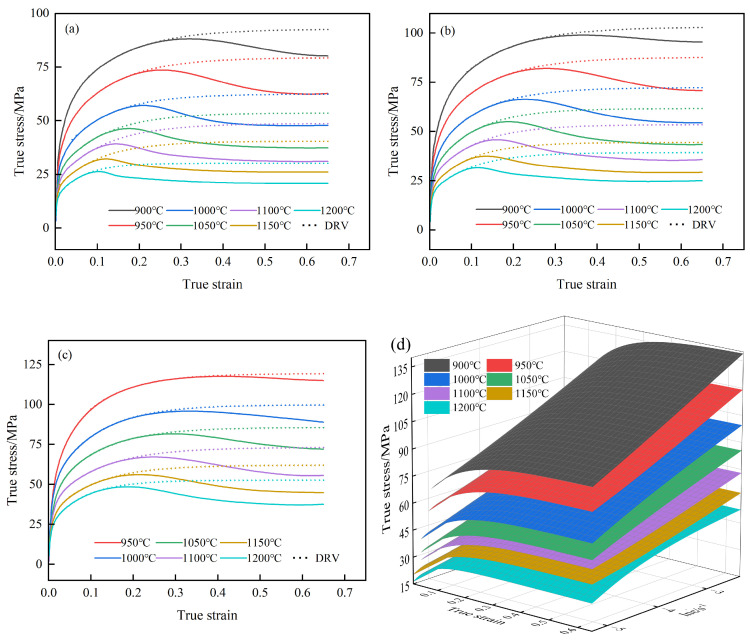
DRV curve and DRX curve of 40CrMnMoA under different conditions: (**a**) 0.005 s^−1^; (**b**) 0.01 s^−1^; (**c**) 0.1 s^−1^; (**d**) 3D response surface graph of recovery stress–strain.

Using Equation (6), the DRV curves of 40CrMnMoA under various conditions were derived, as shown in Figure 6. It can be seen that at the beginning of deformation, the recovery stress rises rapidly with the increase in strain, and then the rise rate decreases but continues to rise until a plateau is reached. At the beginning, the recovery stress is the same as the true stress, and when DRX begins, the recovery curve is higher than the true stress curve. Figure 6d visually shows the effects of temperature, strain and strain rate on recovery stress. The rule is the same as in Section 3.1 and will not be repeated here.

**Figure 7:**
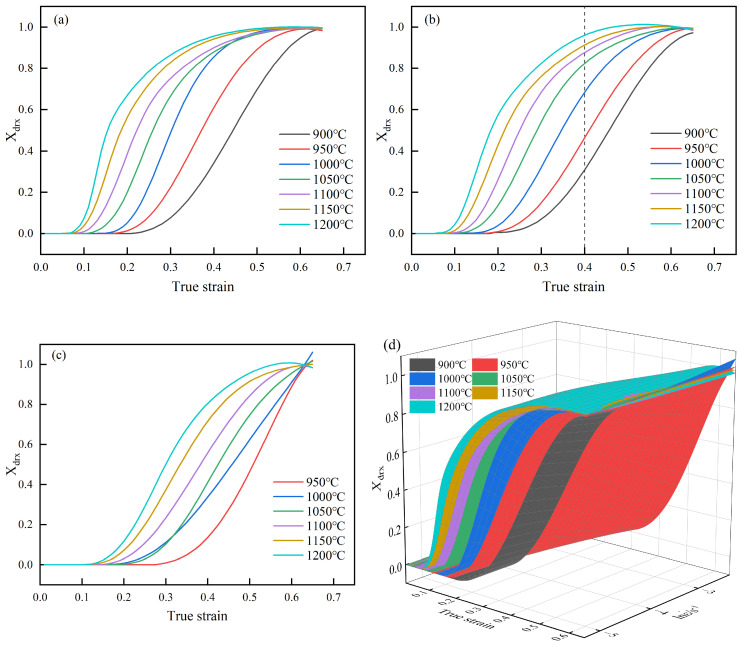
DRX volume fraction curves of 40CrMnMoA under different conditions: (**a**) 0.005 s^−1^; (**b**) 0.01 s^−1^; (**c**) 0.1 s^−1^; (**d**) 3D response surface graph of DRX volume fraction.

By integrating Equation (5) with the data from Figure 6, the dynamic recrystallization fraction ($X_{\mathrm{drx}}$) for 40CrMnMoA under different conditions was calculated and presented in Figure 7. In general, with increasing strain, the percentage of DRX shows an S-curve increasing trend. It can be seen from Figure 7a–c that at fixed strain and strain rate, the percentage of DRX increases with increasing temperature, which can also be seen from Figure 7d. For example, when the strain rate is 0.01s^−1^ and the strain is 0.4, the percentages corresponding to 900, 950, 1000, 1050, 1100, 1150, and 1200 °C are: 31%, 46%, 68%, 83%, 88%, 91%, 96%. In addition, Figure 7d reveals that the percentage of DRX increases as the strain rate decreases.

The characteristic strain $\varepsilon_{0.5}$, defined as the actual strain at which the DRX volume fraction achieves 50% [32], was extracted from Figure 7. The characteristic strains $\varepsilon_{0.5}$ of 40CrMnMoA under various conditions were recorded in Table 3.

The dynamic recrystallization evolution model was first proposed by Sellar et al. [6], and subsequently, different dynamic recrystallization models were proposed by Yada et al. [7] and Kopp et al. [11,12], respectively. Among them, the Kopp model was chosen to characterize the DRX volume fraction of 40CrMnMoA because it can be easily embedded into the commercial FEA software Forge^®^. The Kopp DRX model, which does not consider the initial grain size, is shown in Equation (7):

(7) $$\left\{\begin{array}{l} X_{\mathrm{drx}}=1-\exp\left[-k_{d}{\left(\frac{\varepsilon -\varepsilon_{c}}{\varepsilon_{0.5}-\varepsilon_{c}}\right)}^{n_{\mathrm{drx}}}\right]\\ \varepsilon_{0.5}=A_{0.5}{\dot{\varepsilon }}^{m_{0.5}}\exp(\frac{Q_{0.5}}{RT}) \end{array}\right.$$

In the model, $k_{d}$, $n_{\mathrm{drx}}$, $A_{0.5}$, $m_{0.5}$, $Q_{0.5}$ are physical parameters, $\varepsilon_{0.5}$ is the characteristic strain of 40CrMnMoA alloy steel, and $\varepsilon_{c}$ is the critical strain for the alloy steel to initiate dynamic recrystallization. The traditional method to determine the model parameters is mostly the average method of controlling a single variable. By applying the natural logarithm to Equation (7) and performing the necessary transformations, Equation (8) is derived.

(8) $$\left\{\begin{array}{l} \ln(-\ln(1-X_{\mathrm{drx}}))=\ln k_{d}+n_{\mathrm{drx}}\ln(\frac{\varepsilon -\varepsilon_{c}}{\varepsilon_{0.5}-\varepsilon_{c}})\\ \ln\varepsilon_{0.5}=\ln A_{0.5}+m_{0.5}\ln\dot{\varepsilon }+\frac{Q_{0.5}}{RT} \end{array}\right.$$

**Figure 8:**
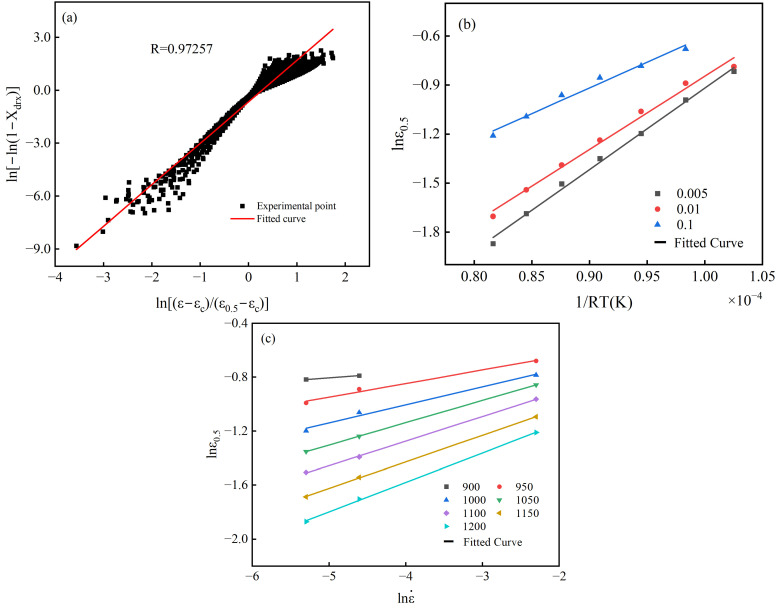
Control single variable method to determine the model parameters: (**a**) $\ln(-\ln(1-X_{\mathrm{drx}}))-\ln[(\varepsilon -\varepsilon_{c})/(\varepsilon_{0.5}-\varepsilon_{c})]$ curve; (**b**) $\ln\varepsilon_{0.5}-1/RT$ curve; (**c**) $\ln\varepsilon_{0.5}-\ln\dot{\varepsilon }$ curve.

According to Equation (8), when $\varepsilon =\varepsilon_{0.5}$ and $X_{\mathrm{drx}}=0.5$, $k_{d}=ln2=0.693$ can be deduced. A plot of $\ln(-\ln(1-X_{\mathrm{drx}}))-\ln[(\varepsilon -\varepsilon_{c})/(\varepsilon_{0.5}-\varepsilon_{c})]$ can be established, where the slope represents $n_{\mathrm{drx}}$, as shown in Figure 8a. In addition, at a constant strain rate, a plot of $\ln\varepsilon_{0.5}-1/RT$ can be created, where *T* is the temperature in Kelvin, and the slope corresponds to $Q_{0.5}$, as depicted in Figure 8b. When the temperature is constant, a plot of $\ln\varepsilon_{0.5}-\ln\dot{\varepsilon }$ can be constructed, with the slope being $m_{0.5}$, as illustrated in Figure 8c. Once $m_{0.5}$ and $Q_{0.5}$ are determined, they can be substituted into Equation (7) to ascertain $A_{0.5}$. The model parameters determined, based on the traditional averaging method, are $n_{\mathrm{drx}}=2.35367$, $Q_{0.5}=42053.11969$ J/mol, $m_{0.5}=0.148662857$, $A_{0.5}=0.012486827$. The determined characteristic strain model is illustrated in Equation (10): 

The critical strain and volume fraction models were obtained using the same methodology, as shown in Equations (9) and (10), respectively.

(9) $$\varepsilon_{c}=0.001961477\times {\dot{\varepsilon }}^{0.183264286}\exp(51682.3123/RT)$$

(10) $$\left\{\begin{array}{l} X_{\mathrm{drx}}=1-\exp\left[-0.693{\left(\frac{\varepsilon -\varepsilon_{c}}{\varepsilon_{0.5}-\varepsilon_{c}}\right)}^{2.35367}\right]\\ \varepsilon_{0.5}=0.012486827{\dot{\varepsilon }}^{0.148662857}\exp\left(\frac{42053.11969}{RT}\right) \end{array}\right.$$

#### 3.3.3. Global Inverse Optimization of DRX Model Parameters

**Figure 9:**
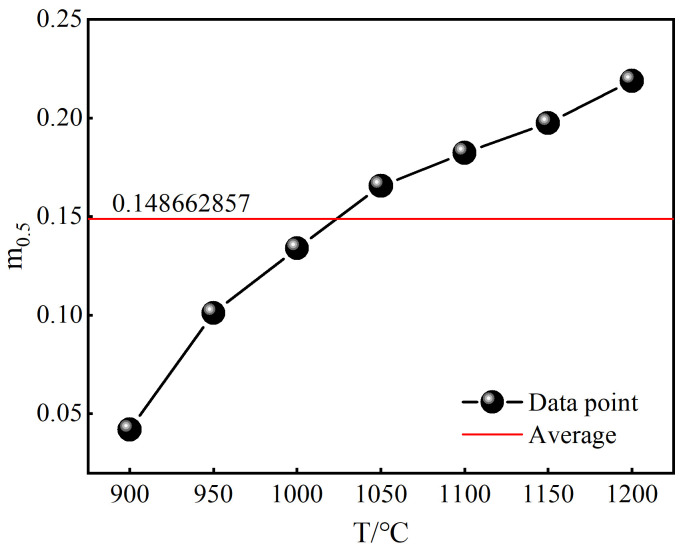
The values of m0.5 under different conditions.

The traditional averaging method is a highly convenient approach for determining model parameters. However, the parameters obtained by this method will fluctuate under different conditions, and the interaction between variables is not taken into account, which limits its accuracy and has certain inherent limitations. For example, when determining the characteristic strain model parameter $m_{0.5}$, the values obtained under different temperatures using the traditional averaging method, along with their mean value, are depicted in Figure 9. The dots represent the $m_{0.5}$ values obtained at 900, 950, 1000, 1050, 1100, 1150, 1200 °C, and the red line represents the average of these values, which is the parameter $m_{0.5}$ in the obtained characteristic strain model. Through Figure 9, it is evident that there is considerable variation in the $m_{0.5}$ values ascertained at different temperatures, with significant discrepancies from the mean value, thereby demonstrating the inherent limitations of the traditional averaging method.

The global optimization algorithm can comprehensively consider the effect of deformation parameters on the model under different conditions. By optimizing the model based on a defined objective function, a more precise model can be achieved. In this study, the ASA algorithm is used to optimize the DRX model of 40CrMnMoA steel, which is a global optimization algorithm that has the advantage of avoiding local optimal solutions and usually has good objective convergence [33].

The objective functions for DRX model optimization were as follows:

(11) $$O(f_{1})=\frac{\sum_{i}^{n}{\left(\varepsilon_{c,i}^{\exp}-\varepsilon_{c,i}^{\mathrm{cal}}\right)}^{2}}{\sum_{i}^{n}{\left(\varepsilon_{c,i}^{\exp}\right)}^{2}}$$

(12) $$O(f_{2})=\frac{\sum_{i}^{n}{\left(\varepsilon_{0.5,i}^{\exp}-\varepsilon_{0.5,i}^{\mathrm{cal}}\right)}^{2}}{\sum_{i}^{n}{\left(\varepsilon_{0.5,i}^{\exp}\right)}^{2}}$$

(13) $$O(f_{3})=\frac{\sum_{i}^{n}{\left(X_{\mathrm{drx},i}^{\exp}-X_{\mathrm{drx},i}^{\mathrm{cal}}\right)}^{2}}{\sum_{i}^{n}{\left(X_{\mathrm{drx},i}^{\exp}\right)}^{2}}$$

where $\varepsilon_{c,i}^{\exp}$, $\varepsilon_{0.5,i}^{\exp}$, $X_{\mathrm{drx},i}^{\exp}$ represent the corresponding critical strain, characteristic strain, and dynamic recrystallization volume fractions under the ith experimental condition, respectively. $\varepsilon_{c,i}^{\mathrm{cal}}$, $\varepsilon_{0.5,i}^{\mathrm{cal}}$, $X_{\mathrm{drx},i}^{\mathrm{cal}}$ denote the critical, characteristic strain, and the DRX volume fraction corresponding to the ith test condition calculated based on Equations (9) and (10), respectively. Therefore, as $X_{\mathrm{drx},i}^{\mathrm{cal}}$ calculated by the model becomes closer to $X_{\mathrm{drx},i}^{\exp}$, $\varepsilon_{c,i}^{\mathrm{cal}}$ becomes closer to $\varepsilon_{c,i}^{\exp}$, and $\varepsilon_{0.5,i}^{\mathrm{cal}}$ becomes closer to $\varepsilon_{0.5,i}^{\exp}$, and the closer the objective function approaches zero, the higher the precision of the model.

**Figure 10:**
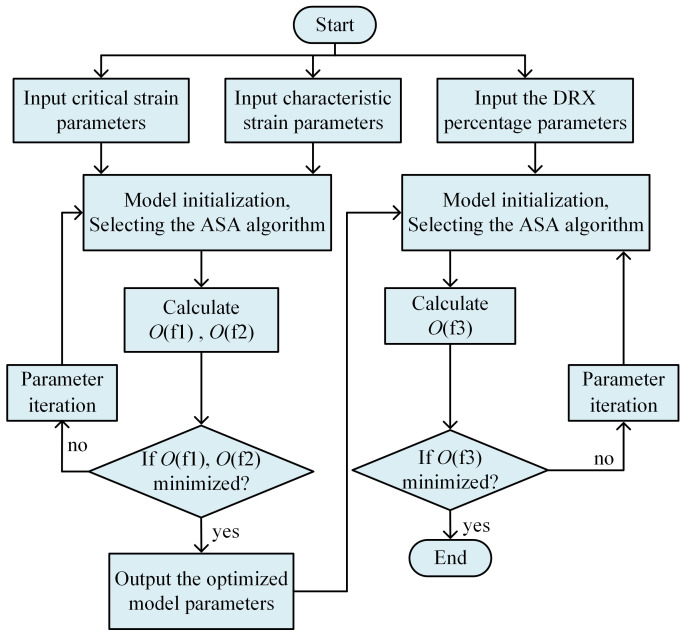
Flowchart for determining DRX model parameters using the inverse optimization method.

The optimization process of the dynamic recrystallization model based on the ASA algorithm is depicted in Figure 10. The process commences with the input of pertinent experimental parameters, such as temperature, strain rate, and the associated critical and characteristic strains. This is followed by an initialization phase, where the critical and characteristic strain models are entered, initial parameter values and their ranges are set, and the ASA algorithm is chosen. The objective functions, $O(f_{1})$ and $O(f_{2})$, are then computed. It is then ascertained whether these functions were minimized; if they are, the current model parameters are output. If not, adjustments are made to the parameters, and the functions are re-evaluated. The optimization of the critical strain and characteristic strain model parameters are conducted in tandem. Post-optimization of these parameters, they are introduced into the DRX volume fraction model’s optimization sequence, adhering to the same procedure for further optimization.

The initial values for the critical strain model and DRX model parameters of 40CrMnMoA steel are obtained using the traditional averaging method, with the value range adjusted above and below the initial value. A larger range can be initially chosen, followed by multiple adjustments. The initial values and parameter range for each model used in the ASA algorithm are shown in Table 4.

**Figure 11:**
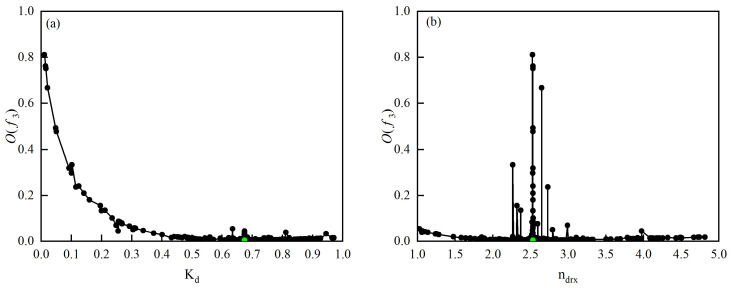
The influence of different model parameters on the objective function. (**a**) $O\left(f_{3}\right)-K_{d}$ (**b**) $O\left(f_{3}\right)-n_{\mathrm{drx}}$ curve.

Figure 11 shows the impact of different model parameters on the objective function $O(f_{3})$. The curve indicates that $O(f_{3})$ is minimized when the $K_{d}=0.674240973$ and $n_{\mathrm{drx}}=2.53289839$ (green dots in Figure 11), suggesting that the model for the dynamic recrystallization volume fraction is the most accurate at these parameter values. The ultimately determined dynamic recrystallization model for 40CrMnMoA alloy steel is as follows:

(14) $$\varepsilon_{c}=0.001424221\times {\dot{\varepsilon }}^{0.160832156}\exp\left(\frac{54012.17267}{RT}\right)$$

(15) $$\left\{\begin{array}{c} X_{\mathrm{drx}}=1-\exp\left(-0.674240973{\left(\frac{\varepsilon -\varepsilon_{c}}{\varepsilon_{0.5}-\varepsilon_{c}}\right)}^{2.53289839}\right)\\ \varepsilon_{0.5}=0.014641609\times {\dot{\varepsilon }}^{0.144306904}\exp\left(\frac{40211.35229}{RT}\right) \end{array}\right.$$

The established percentage model can predict the dynamic recrystallization percentage under different conditions. To reflect the accuracy of the optimized model, the correlation coefficient *R*, mean absolute relative error (*AARE*), and root-mean-square error (*RMSE*) were used [34], as detailed in Equations (16)–(18).

(16) $$R=\frac{\sum_{i=1}^{n}\left(y_{e}^{i}-{\bar{y}}_{e}\right)\left(y_{p}^{i}-{\bar{y}}_{p}\right)}{\sqrt{\sum_{i=1}^{n}{\left(y_{e}^{i}-{\bar{y}}_{e}\right)}^{2}}\sqrt{\sum_{i=1}^{n}{\left(y_{p}^{i}-{\bar{y}}_{p}\right)}^{2}}}$$

(17) $$RMSE=\sqrt{\frac{1}{n}\sum_{i=1}^{n}{\left(y_{e}^{i}-y_{p}^{i}\right)}^{2}}$$

(18) $$AARE\left(\%\right)=\frac{1}{n}\sum_{i=1}^{n}\left|\frac{y_{e}^{i}-y_{p}^{i}}{y_{e}^{i}}\right|\times 100$$

In the formulas: $y_{e}$ represents the percentage obtained from experiments; $y_{p}$ represents the percentage calculated by the model; ${\bar{y}}_{e}$ and ${\bar{y}}_{p}$ p are the average values of $y_{e}$ and $y_{p}$, respectively; *i* denotes the ith data point; n is the total number of data points.

**Figure 12:**
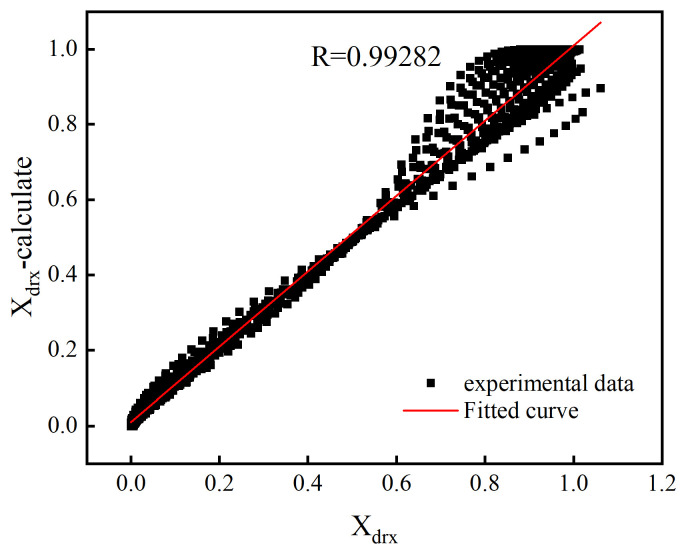
The correlation of predicted and experimental values of dynamic recrystallization percentage.

Based on Equations (16)–(18), the values of *R*, *AARE*, and *RMSE* of the DRX percentage model before and after optimization were calculated and recorded in Table 5. It indicates that after optimization by the ASA algorithm, the *R*-value improved from 0.925 to 0.992; concurrently, the *AARE* and *RMSE* were reduced from 92.131 and 0.047 pre-optimization to 60.945 and 0.046 post-optimization. Figure 12 presents a comparison of the predicted DRX percentages with experimental data, demonstrating a significant correlation. Therefore, the model optimized by the adaptive simulated annealing algorithm can more accurately describe the DRX behavior of 40CrMnMoA alloy steel.

#### 3.3.4. Validation of DRX Model for 40CrMnMoA Steel

To verify the accuracy of the established models, the determined DRX model was first embedded into FEA software Forge^®^ 3.2. Then, a simulation of the isothermal hot compression process of 40CrMnMoA under conditions of 0.01 s^−1^ was conducted. During the simulation, the descending speed of the press was configured at 0.09 mm/s, with a compression extent of 6 mm.

**Figure 13:**
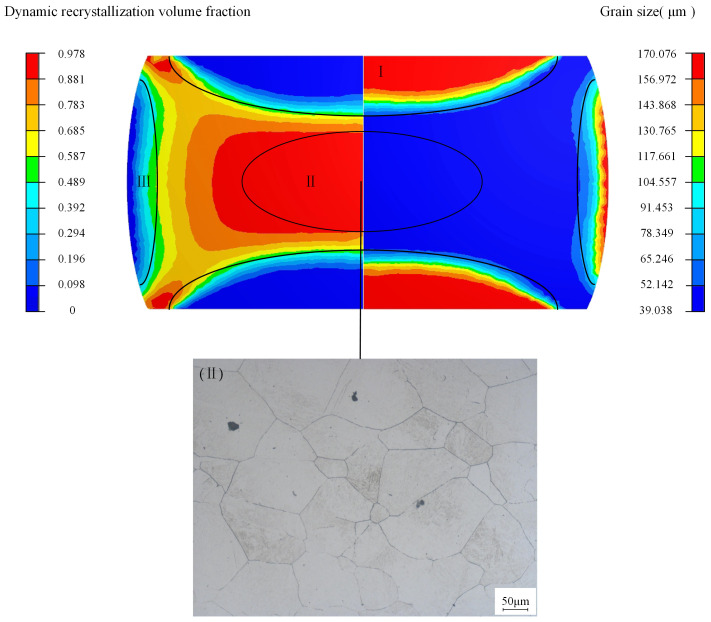
Comparison between simulation results and compression experiment in large deformation region (II) at 1100 °C, 0.01 s^−1^.

Figure 13 shows the simulation results of isothermal compression of cylindrical specimens at 1100 °C and 0.01 s^−1^. On the left is the cloud plot depicting the DRX volume fraction, while the right displays the grain size distribution. Below these is the metallographic image of the central region of the sample under corresponding conditions. The deformed specimen can be broadly divided into three areas: hard deformation region (I), large deformation region (II), and small deformation region (III). Due to limited deformation in Zones I and III, dynamic recrystallization is incomplete, so the discussion here focuses on the significantly deformed area. At the core of the cylindrical specimen (Zone II), the dynamic recrystallization volume fraction is the highest, reaching 0.978, with a grain size of 42.331 μm. The metallographic analysis reveals a grain size of 45.256 μm, yielding a relative discrepancy of 6%.

**Figure 14:**
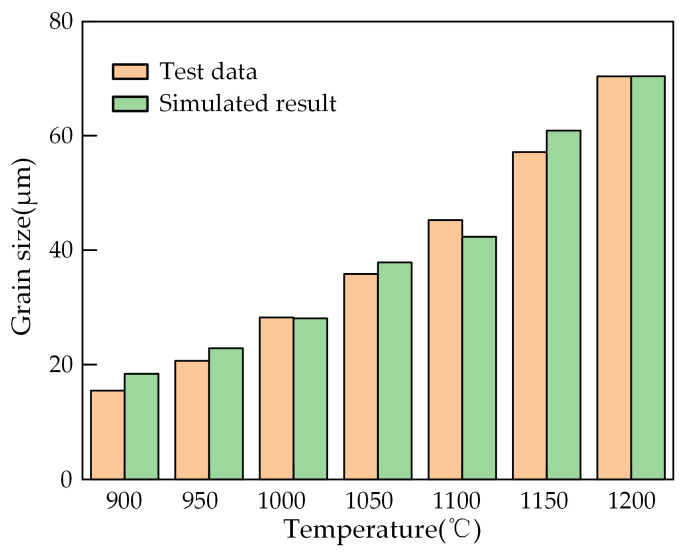
Comparison between simulated and tested grain size under 0.01 s^−1^ condition.

The simulation of the compression process of 40CrMnMoA was calculated at 900, 950, 1000, 1050, 1150 and 1200 °C at a strain rate of 0.01s^−1^. The grain size in the central region of the simulated sample was compared with that in the central region of the metallographic photo, as shown in Figure 14, and the relative error was 3%. This indicates that the established DRX model possesses a high level of prediction accuracy and is suitable for predicting the DRX behavior of 40CrMnMoA alloy steel, and further proves the effectiveness of the inverse optimization method used.

## 4. Conclusions

An inverse optimization method was proposed to calculate the DRX model parameters of 40CrMnMoA in this work. The optimized DRX model was then embedded into the FEA software Forge^®^, followed by a simulation of the hot compression process. The grain size from the central region of specimens was contrasted with simulation outcomes, thereby verifying the feasibility of the inverse optimization method. The principal conclusions can be drawn as follows:

1. Drawing from the true stress–strain data of 40CrMnMoA steel, its DRX behavior was analyzed. The steel tends to exhibit DRX behavior at high temperatures and low strain rates, especially at 1200 °C, 0.005 s^−1^. In contrast, at lower temperatures and higher strain rates, such as 950 °C, 0.1 s^−1^, DRX is either absent or minimal.
2. Utilizing the inverse optimization method, the DRX model for 40CrMnMoA steel was established, achieving *R*, *AARE*, and *RMSE* values of 0.992, 60.945, and 0.046, respectively. These results confirmed the high precision of the developed model.
3. Through the user subroutine, the determined DRX model was successfully embedded into the FEA software Forge^®^. The isothermal compression process was simulated at a strain rate of 0.01s^−1^ across various temperatures and cloud plots of DRX volume fraction and grain size were obtained. The grain size in the central region of the simulation results closely matched that of the experimental samples. The results indicate that the inverse optimization method can accurately identify the DRX model parameters of 40CrMnMoA alloy steel.
4. The DRX model established based on the inverse optimization method reveals the dynamic recrystallization evolution of 40CrMnMoA steel more accurately, which is of great scientific value for the microstructure prediction and process design of large extrusion hot roll forming.

## Figures and Tables

**Table 1:** Chemical composition of 40CrMnMoA steel (mass fraction, %).

C	Mn	Si	P	S	Ni	Cr	Mo	Fe
0.42	1.0	0.36	0.007	0.005	0.25	1.1	0.3	Bal

**Table 2:** Peak strain $\varepsilon_{p}$ and critical strain $\varepsilon_{c}$ of 40CrMnMoA alloy steel.

Temperature	0.005 s^−1^		0.01 s^−1^		0.1 s^−1^	
	$\varepsilon_{c}$	$\varepsilon_{p}$	$\varepsilon_{c}$	$\varepsilon_{p}$	$\varepsilon_{c}$	$\varepsilon_{p}$
900 °C	0.157	0.319	0.177	0.367	-	-
950 °C	0.123	0.254	0.135	0.279	0.201	0.412
1000 °C	0.102	0.209	0.110	0.227	0.144	0.332
1050 °C	0.082	0.175	0.087	0.188	0.142	0.295
1100 °C	0.068	0.145	0.076	0.16	0.117	0.249
1150 °C	0.057	0.12	0.063	0.135	0.101	0.214
1200 °C	0.049	0.103	0.056	0.115	0.085	0.187

**Table 3:** Characteristic strains of 40CrMnMoA under various conditions.

Temperature	0.005 s^−1^	0.01 s^−1^	0.1 s^−1^
900 °C	0.442	0.455	-
950 °C	0.371	0.411	0.507
1000 °C	0.302	0.346	0.457
1050 °C	0.259	0.29	0.425
1100 °C	0.222	0.249	0.382
1150 °C	0.185	0.214	0.335
1200 °C	0.154	0.182	0.298

**Table 4:** Initial values and ranges for DRX model parameters of 40CrMnMoA alloy steel.

Parameters	Initial Value	Range	Parameters	Initial Value	Range
$A_{c}$	0.001961477	0~0.05	$n_{1}$	1	0.5~1
$m_{c}$	0.183264286	0~1	$n_{2}$	1	0.5~1
$Q_{c}$	51,682.3123	10,000~100,000	$n_{\mathrm{drx}}$	2.35367	1~5
$A_{0.5}$	0.012486827	0~0.5	$A_{d}$	45,822.75598	10,000~100,000
$m_{0.5}$	0.148662857	0~1	$m_{d}$	0.117188571	0~1
$Q_{0.5}$	42,053.11969	10,000~100,000	$Q_{d}$	−72,714.96784	−10,000~−100,000
$k_{d}$	0.693	0~1			

**Table 5:** Comparison of various indicators before and after optimization.

	*R*	*RMSE*	*AARE*/%
Traditional method	0.925	0.047	92.131
ASA optimization	0.992	0.046	60.945

## Data Availability

The data presented in this study are available on request from the corresponding author. The data are not publicly available as these data are part of ongoing research.
